# New dicoumarol sodium compound: crystal structure, theoretical study and tumoricidal activity against osteoblast cancer cells

**DOI:** 10.1186/1752-153X-7-110

**Published:** 2013-07-02

**Authors:** Sadia Rehman, Muhammad Ikram, Ajmal Khan, Soyoung Min, Effat Azad, Thomas S Hofer, KH Mok, Robert J Baker, Alexander J Blake, Saeed Ur Rehman

**Affiliations:** 1 Institute of Chemical Sciences, University of Peshawar, Peshawar, Pakistan; 2 Department of Chemistry, Sarhad University of Science and Information Technology, Peshawar, Pakistan; 3 Institute of General, Inorganic and Theoretical Chemistry, University of Innsbruck Innrain 80–82, A-6020 Innsbruck, Austria; 4 Trinity Biomedical Sciences Institute (TBSI) School of Biochemistry and Immunology Trinity College, Dublin 2, Ireland; 5 School of Chemistry, University of Dublin Trinity College, Dublin 2, Ireland; 6 School of Chemistry, The University of Nottingham, Nottingham NG7 2RD, UK

**Keywords:** Dicoumarol, Sodium cation, Single crystal study, DFT, Bone cancer

## Abstract

**Background:**

Enormous interest had been paid to the coordination chemistry of alkali and alkaline metal ions because of their role inside body viz; their Li^+^/Na^+^ exchange inside the cell lead to different diseases like neuropathy, hypertension, microalbuminuria, cardiac and vascular hypertrophy, obesity, and insulin resistance. It has been presumed that alkali metal ions (whether Na^+^ or K^+^) coordinated to chelating ligands can cross the hydrophobic cell membrane easily and can function effectively for depolarizing the ion difference. This unique function was utilized for bacterial cell death in which K^+^ has been found coordinated valinomycin (antibiotic).

**Results:**

Distinct sodium adduct (**1**) with dicoumarol ligand, 4-Hydroxy-3-[(4-hydroxy-2-oxo-4a,8a-dihydro-2H-chromen-3-yl)-phenyl-methyl]-chromen-2-one (**L**) is isolated from the saturated solution of sodium methoxide. Single crystal X-ray diffraction studies of the adduct reveals that sodium is in the form of cation attached to a methoxide, methanol and a dicoumarol ligand where carbonyl functional groups of the coumarin derivative are acting as bridges. The sodium compound (**1**) is also characterized by IR, ^1^H-NMR, and ^13^C{^1^H}-NMR spectroscopic techniques. The composition is confirmed by elemental analysis. DFT study for **1** has been carried out using B3LYP/6-13G calculations which shown the theoretical confirmation of the various bond lengths and bond angles. Both the compounds were studied subsequently for the U2OS tumoricidal activity and it was found that **L** has LD_50_ value of 200 μM whereas the sodium analog cytotoxicity did not drop down below 60%.

**Conclusion:**

A sodium analogue (**1**) with medicinally important dicoumarol ligand (**L**) has been reported. The crystal structure and DFT study confirm the formation of cationic sodium compound with dicoumarol. The ligand was found more active than the sodium analog attributed to the instability of **1** in solution state. Coumarin compound with sodium was observed to be less cytotoxic than the ligand, its LD_50_ value never dropped below 60%.

## Findings

This work is dealing with the synthesis of sodium derivative of dicoumarols. The structure was assigned based upon the single crystal diffraction and DFT studies. It was presumed that the novel sodium compound will show very good results in biological system but the opposite behavior was observed owing to the instability of the compound in solution phase. We are searching for the possible answers in this unique research and soon will be available to the reader.

## Background

In malignant sarcoma the neoplasm of the malignant tissues produces malignant osteoid which causes bone cancer. This is prevailing 4 in 100,000 people/year and is a leading cancer disease in children’s under age 15. Leg or hand amputation, a disaster to the human or animal body, is caused by osteosarcoma [1].

**Scheme 1:**
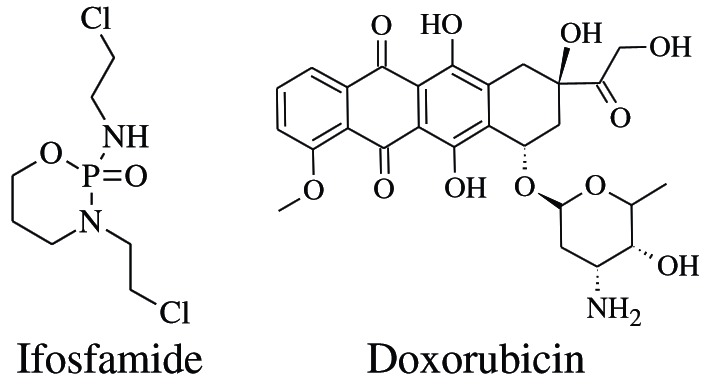
Drugs for the treatment of synovial sarcoma.

There are different types of sarcomas including the synovial sarcoma, Ewing's sarcoma and osteosarcoma which are more often found in young adults and neoplasias such as leiomyosarcoma or liposarcoma are found in humans of more than 55 years age [1,2]. Synovial sarcoma is being treated using doxorubicin and ifosfamide as shown in Scheme 1[3].

The doxorubicin has been found to cause neutropenia, alopecia, dispigmentation, and reactivation of Hepatitis B, cardiomyopathy or even death. Therefore due to its very toxic nature it had been named as “Red Devil” [4-7]. Similarly ifosfamide causes encephalopathy (brain dysfunction), a very serious drawback of the drug. Encephalopathy is actually caused by the production of toxic side products like acetaldehyde and chloral hydrate [8].

Coumarin and its derivatives are biologically very active. It was found that the enhanced activities are dependent on the coumarin nucleus [8-12]. Biological significance of these compounds include anti-bacterial [12] anti-thrombotic and vasodilatory [13], anti-mutagenic [14] lipoxygenase and cyclooxygenase inhibition [15,16], scavenging of reactive oxygen species, and anti-tumourigenic [17-23]. The fact that such compounds have medicinal applications, prompted many researchers to work in this field and several recent reviews summarize advances in this field [24-29]. Coumarin and its derivatives have been applied for treatment of different types of cancers including malignant melanoma, leukaemia, renal cell carcinoma, prostate and breast cancer. Coumarin when applied in combination with radiotherapy and surgery as a chemotherapeutic agent provides best results. It, not only treat cancer but also decreases the side effects of radio therapy [30].

Coumarin and its derivatives have also been used in the treatment of Malignant Melanoma [23]. Initially the melanoma diagnosis involved surgical removal of primary lesion with the high risk of recurrence after five years. However the problem was minimized by the use of 4-hydroxycoumarin along with warfarin to maintain therapy and to inhibit the tumor spread. Therefore, 4-hydroxycoumarins have been successful in adjuvant therapy for melanoma [31,32].

Coumarin and its derivatives have been investigated for their possible use in the treatment of renal cell cancer [33,34]. In vitro effects of such compounds on the growth of renal cell carcinoma derived cell lines proved coumarin and 7-hydroxycoumarin as potent cytotoxic and cytostatic agent [35]. Similarly in case of other types, like leukaemia, prostate and breast cancer, cyclin D_1_ is released in an amount more than the normal levels. Coumarin and its derivatives have been proved significant antiproliferative agents by regulating the release of Cyclin D_1_[36-38].

Enormous interest had been paid to the coordination chemistry of alkali and alkaline metal ions because of their role inside body viz; their Li^+^/Na^+^ exchange inside the cell lead to different diseases like neuropathy, hypertension, microalbuminuria, cardiac and vascular hypertrophy, obesity, and insulin resistance. It has been presumed that alkali metal ions (whether Na^+^ or K^+^) coordinated to chelating ligands can cross the hydrophobic cell membrane easily and can function effectively for depolarizing the ion difference. This unique function was utilized for bacterial cell death in which K^+^ has been found coordinated valinomycin (antibiotic). Alkali and alkaline metals ions complexed with many coordinating ligands like crown ethers, cryptands, pyrazolyl, nitriles, phosphanide and many others were studied previously [39-45].

In the recent report we have focused upon the modified coumarin and its sodium derivative, keeping in mind the aforementioned importance of coumarin nucleus. The modified coumarin has been prepared by reacting bezaldehyde with 4-hydroxycoumarin to yield the dicoumarol ligand. Efforts for deprotonating the hydroxyl groups, using the sodium methoxide, gave the disodium coumarin compound. The sodacation compounds has been studied structurally by X-ray crystallography, supported by DFT calculations and spectroanalytical techniques. The compounds were screened, In Vitro, for U2OS anticancer activities, presuming sodium derivatives will be more active than the parent ligand. However, the observed results were contradictory to our assumption, probably, due to the instability of sodium compound inside cell matrix. This aspect of the sodium compound may be further studied in detail to see the actual possible reason behind the presumed bahvior.

## Results and Discussion

**Figure 1:**
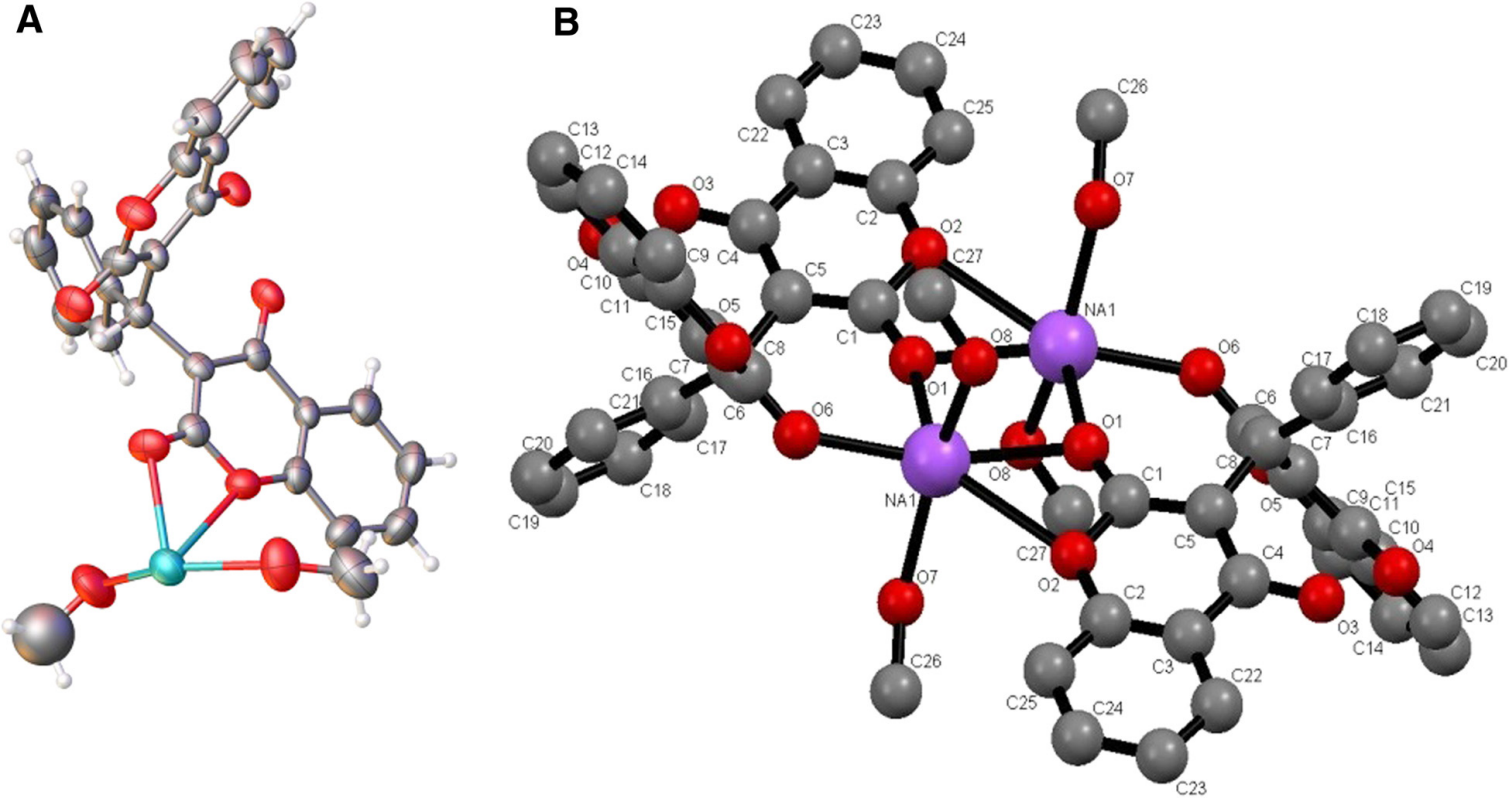
**Crystal structure of 1. A)** Crystal structure of 1. Crystal Data., monoclinic, a = 12.3792(14) Å, b = 11.5396(7) Å, c = 16.980(2) Å, β = 102.354(12)°, V = 2369.4(4) Å3, T = 298 K, space group P21/n (no. 14), Z = 1, μ(N/A) = N/A, 7498 reflections measured, 4233 unique (R_int_ = 0.1007) which were used in all calculations. The final wR_2_ was 0.200383 (all data) and R_1_ was 0.116317 (I > 2u(I)). **B)** Labelled diagram for NMR assignment of 1.

**Figure 2:**
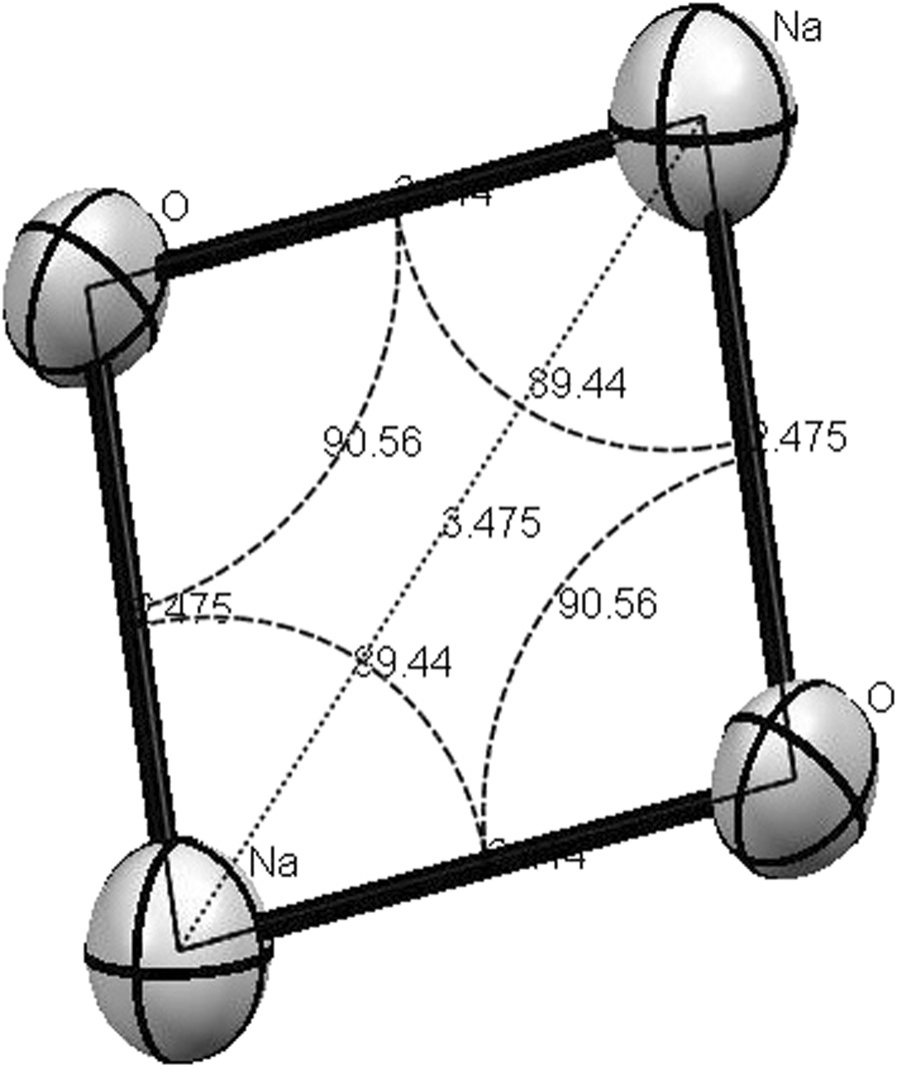
**Section of the crystal lattice of 1 showing Na**_**2**_**O**_**2 **_**bridge.**

Crystal structure of **1**, as shown in Figure 1 whereas Figure 2 represents the fragment of the sodium adduct with **L**.

It is revealed by looking into the crystal structure of **1** that sodium cations are attached to the carbonyl oxygen O(6)-Na at distance 2.414 Å while the bond length for sodium cation attached to carbonyl and lactone oxygen O(1)-Na-O(2) is 2.475 Å showing trans tetragonal planar arrangement of the atoms in space. This behaviour is further proved by the bond angles pattern of the same Na_2_O_2_ plane where the bond angle of O(1)-Na(1)-O(1) is 89.44^o^ while the bond angle for Na(1)-O(1)-Na(1) is 90.56^o^ revealing coplanarity of the structure. Inter cationic distance between the two sodium cations is 3.441 Å. The bond distance for C = O for the C(1)-O(1) in **1** is 1.247 Å while for C(8)-O(6) is 1.199 Å, the former is 0.048 Å longer than the latter which may be attributed to the bond formation from the lactone oxygen. The bond distance for O(1)-Na(1) is 2.873 Å suggesting that the bond is weaker. By comparing the bond angles of O(7)-C(1)-O(1) and O(8)-C(8)-O(6) which are 112.5^o^ and 114.59^o^ respectively, suggest that C = O {C(1)-O(1)}angle is decreased by the bond formation between O(1) and Na(1). The bond distance Na(1)-O(8) is 2.350 Å identical for all the methoxide/methanol ions attached to the sodium. Sodium cation produces an eight membered ring with O(6)-C(8)-C(7)-C(6)-C(5)-C(1)-O(1) atoms. The plane produced by Na-O-Na-O is separated by 20.04^o^ from the plane produced by C(8)-C(7)-C(5)-C(1)-Na(1). If C(6) is included in the plane produced by chelate ring then the two planes are separated by a mean angle of 17.89^o^. Therefore, it shows that C(6), due to the attached phenyl ring, does not lie in the plane produced by C(8)-C(7)-C(5)-C(1)-Na(1). The DFT structural features were also studied.

**Figure 3:**
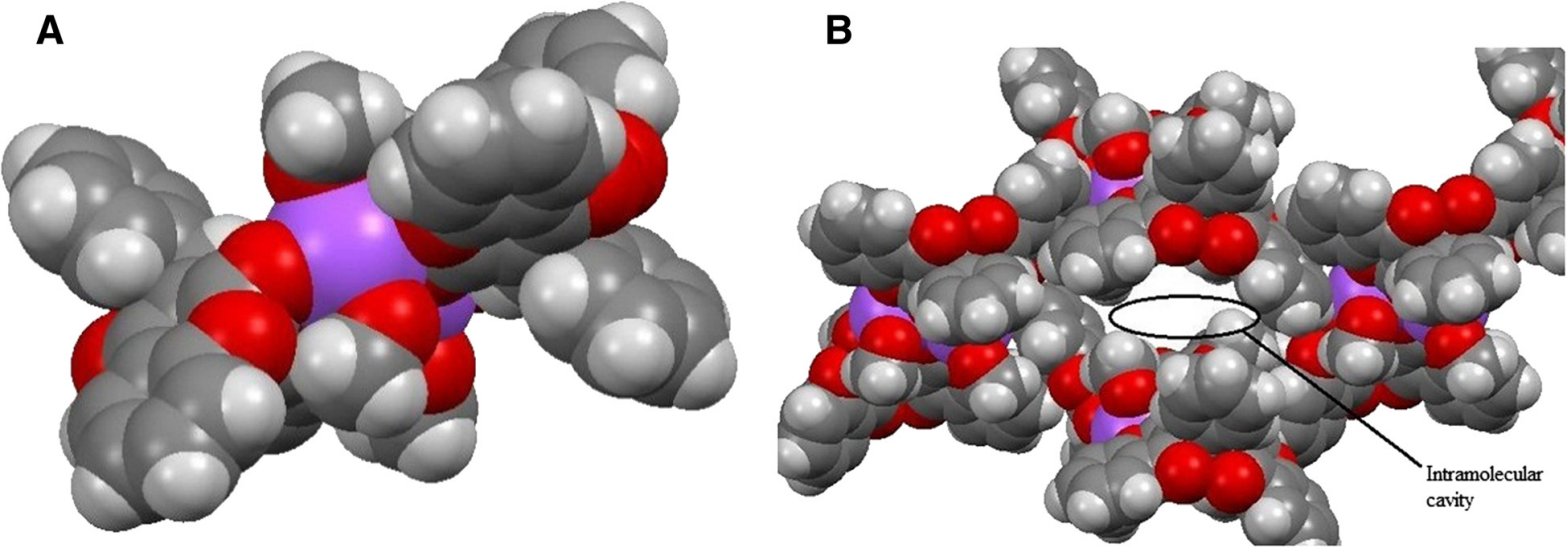
**Space filled model of 1. A)** Space filled model of 1. It has been shown here that sodium ions are embedded by dicoumarol ligands **B)** Wide geometric cavity.

Figure 3(A) clearly show the ligands coordination to the sodium cations. The hydrophobic sites are protruding out making it possible for the sodium compound to cross the hydrophobic cell membrane. Large cavities can be seen in Figure 3B.

The IR spectrum of the ligand was assigned, the carbonyl stretching frequency in ligand was observed around 1647 cm^-1^ which may be due to hydrogen bonding [20] and it was displaced to 1671 cm^-1^ after complexation with sodium. The C-O phenolic vibration observed at 1303 cm^-1^ was also displaced to 1349 cm^-1^ by coordination with sodium.

^1^H and ^13^C{^1^H}-NMR results are also consistent with the structures obtained. ^1^H-NMR in DMSO-*d*_*6*_ shows a singlet at 6.10 ppm which was assigned to –CH(Ar)_2_ peak, whereas the aromatic protons are observed in the range of 7.20-7.90 ppm. The hydroxyl protons were not observed in the sodium complex. In ^13^C{^1^H}-NMR the peak for the CH_3_ of the methoxide ions is observed at 103 ppm because of coordination to sodium cations. Rest of the spectrum is almost identical to **L**. ^23^Na-NMR was also recorded for the compound which shows a single peak at −1.01 ppm suggesting the presence of sodium cation.

**Figure 4:**
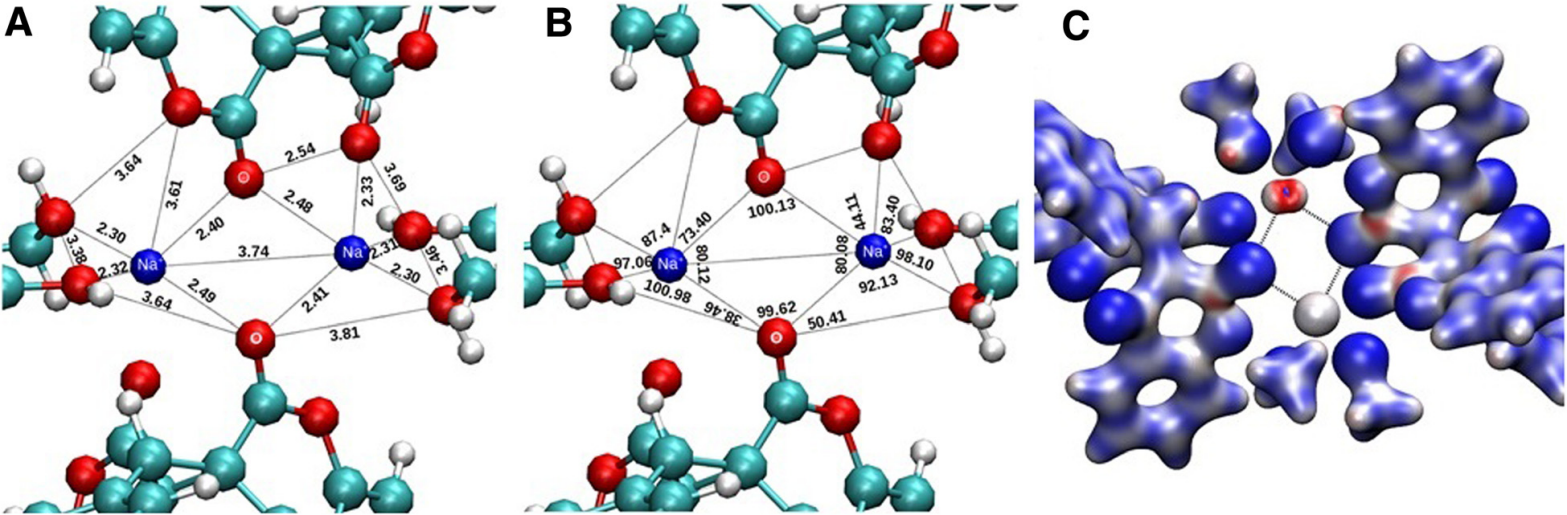
**Calculated bond lengths and angles of 1. (A)** Interatomic distances in Å calculated at the B3LYP/6-31G (d, p) level **(B)** Interatomic angles in degree calculated at the B3LYP/6-31G (d, p) level **(C)** Electrostatic potential projected on isodenisty surface computed at the B3LYP/6-13G (d, p) level highlighting regions with varying electron density.

Molecular ion peak was not observed in DMSO. Therefore elemental analysis was carried out which revealed the mentioned composition. The molar conductance value suggest the presence of free ions in the solution state, therefore it was concluded that the complex ionizes when dissolved. The DFT structural study had been carried out using Gaussian G09 at B3LYP/6-13G (d, p) level [46]. The minimum energy calculated from geometric optimization of Gaussian G09 at B3LYP/6-13G (d, p) level is −3612 Hatree. The geometrical optimized structure from Gaussian is similar to the single crystal structure in term of interatomic distances as well as in angles (Figure 4A and Figure 4B).

The theoretical structure for **1** was optimised inorder to get into the conclusion of correct structure for **1**. The difference between the calculated and the observed bond lengths and angles has been shown in Table 1, revealing that there is not much difference for the bond lengths but the bond angles are not so close, therefore it can be concluded that one of the methoxide ions is acting as neutral methanol molecule.

**Table 1:** Comparison of the calculated vs. experimental bond lengths and bond angles for compound 1

**Moiety**	**Theor. bond length, A**^**o**^	**Exp. bond length, A**^**o**^	**Moiety**	**Theor. bond angle **^**o**^	**Exp. bond angle **^**o**^
Na-O (coumarin)	2.40, 2.48	2.41, 2.47	O-Na-O	80.12	89.44
Na-O (methanolic)	2.32	2.31	Na-O (methanolic)	97.08, 100.98	100.56, 106.33

The electrostatic potential projected on isodensity surface computed at B3LYP/6-31G /d, p) is depicted in Figure 4C, highlighting regions with varying electron density.

**Figure 5:**
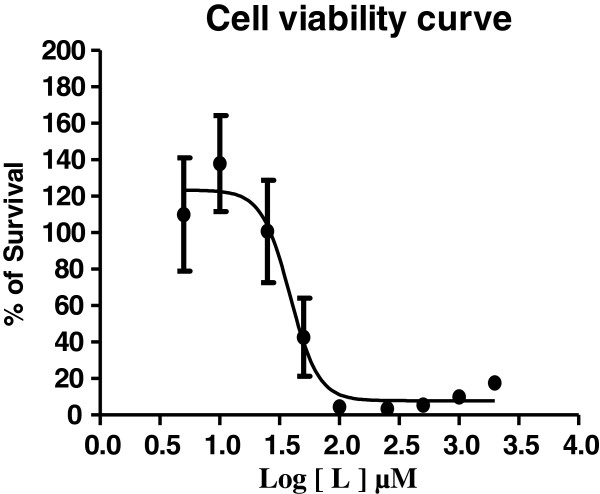
**Dose response curve of L.** Treatment of U2OS cells with different concentrations of L compound and its cytotoxicity as observed with Alamar Blue.

**Figure 6:**
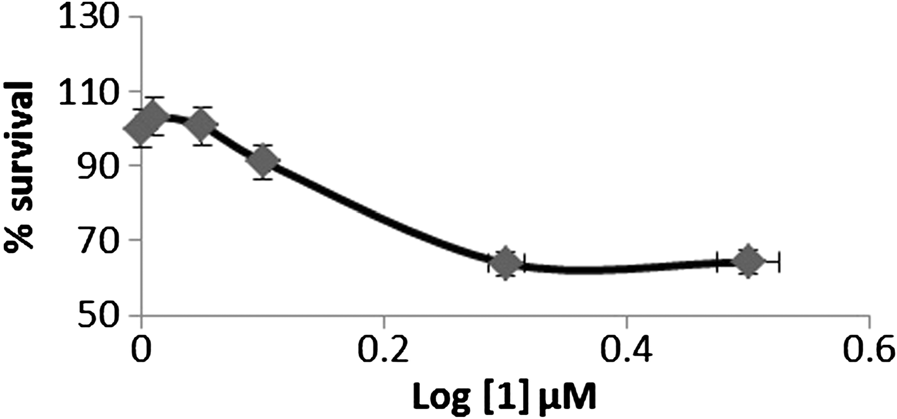
**Dose response curve of 1.** Treatment of U2-OS cells with different concentrations of 1 compound and its cytotoxicity as observed Alamar Blue.

The ligand **L** and sodium analog of **L** were studied In Vitro for the cytotoxic activities against osteoblast U2OS cancerous cells and it was found that **L** exhibited a well-behaved cytotoxicity curve (with an LD_50_ value of 200 μM; Figure 5), for the concentration range tested with regards to **1**, the cytotoxicity survival values never dropped below 60% (Figure 6). Although the testing of higher concentrations of **1** is needed, it may be the case where the structural changes induced by the coordination with sodium may be influencing the cytotoxicity *in vivo*.

## Experimental

### Materials and Methods

**Scheme 2:**
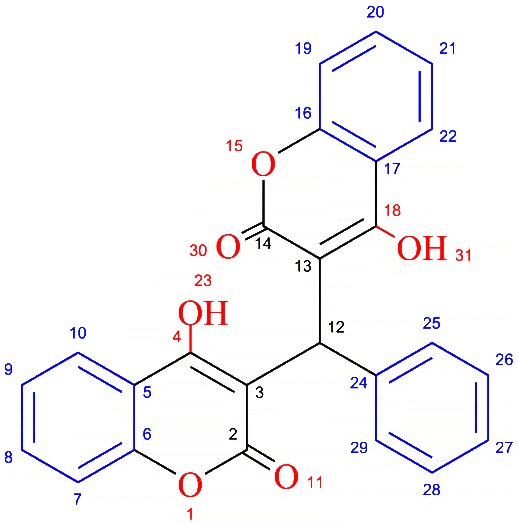
Atom numbering for NMR assignment of L.

All the manupulations were performed under argon environment using Schlenk line system. 4-hydroxycoumarin (fluka), and benzaldehyde (Sigma Aldrich) were used as received. Analytical grade methanol (Aldrich) was dehydrated and degassed prior to use. Sodium metal (Fluka) was added to methanol to produce sodium methoxide under argon. Elemental analyses were carried out on Varian Elementar II. FT-IR spectra were recorded using Shimadzo FTIR Spectrophotometer Prestige-21. ^1^H-NMR was measured with Bruker DPX 400 MHz (400.23 MHz) whereas, ^13^C{^1^H}NMR was recorded on Bruker AV 400 MHz (150.9 MHz) spectrometers in DMSO-*d*^*6*^ at room temperature. Chemical shifts were reported in ppm and standardized by observing signals for residual protons. Chemical resonances for **L** were assigned using structure shown in Scheme 2 and for **1** the resonances were assigned using Figure 1B. Mass spectra were recorded on a LCT Orthogonal Acceleration TOF Electrospray mass spectrometer. Single crystal analyses were carried out using oxford diffractometer. Suitable single crystals for X-ray structural analyses of 1 was mounted on a glass fibre, and the respective data were collected on the diffractometer (graphite-monochromated Mo Kα radiation, λ = 0.71073 Å) at 108(2) K. The structures were solved with the olex2.solve [47] structure solution program using Charge Flipping and refined with the olex2.refine [48] refinement package using Gauss-Newton minimisation. Crystallographic details are given in the Additional file 1. CCDC-868924 (1) data can be obtained free of charge from the Cambridge Crystallographic Data Centre via http://www.ccdc.cam.ac.uk/data_request/cif.

The geometric optimization was carried out at the restricted B3LYP [49] hybrid density functional level, as implemented in the G09 program [46]. The X-ray structure was employed as starting configuration for the geometric optimization. The 6-31 g(d,p) basis set was assigned to all atoms [50].

### Cytotoxic activity

The human osteosarcoma cell line U2OS was used for testing the tumoricidal activities of the **1** and **L** compounds. The cells were cultured in DMEM + GlutaMAXTM-1 with added 1% penicillin and streptomycin and 10% heat-inactivated fetal bovine serum. For adherent cells, trypsin-EDTA was used for detachment. The cells were washed in Dulbecco’s phosphate buffered saline (DPBS), harvested by centrifugation (1000 rpm, 5 min), and resuspended in DMEM. The cells were seeded into 96-well plates at a density of 5×10^3^ cells / well, and incubated at 37°C in 5% CO_2_ atmosphere for 24 hours before being treated with the compounds. The compounds were initially dissolved at a stock concentration of 10 mM in DMSO and added to wells to a final concentration between 5.0 and 2000 μM. The plates were then incubated at 37°C for a further 24 hours for treatment. Alamar blue (Invitrogen) was used to test the viability of the cells (10 μl per well). Plates in triplicate were incubated for 4 hours at 37°C protected from direct light, then read at 590 nm using an excitation wavelength of 544 nm in a fluorescence plate reader (Spectra Max Gemini). Wells containing medium and distilled water-only served as blank controls, while the viability of the treated cells was taken as a percentage compared to wells with untreated cells. The LD_50_ value of each compound was estimated by fitting the correlation between cell viability and compound concentration.

#### Synthesis of 4-Hydroxy-3-[(4-hydroxy-2-oxo-4a,8a-dihydro-2H-chromen-3-yl)-phenyl-methyl]-chromen-2-one (L)

The dicoumarol ligand (**L**) was synthesized by the reported procedure [51], 25 mmol benazldehyde was added to the 50 mmol stirred ethanolic solution of 4-hydroxycoumarin and the mixture was refluxed for 3 hr at 80°C. After cooling the reaction mixture, solid white powder of the **L** were isolated, washed several times with copious 10% ethanolic n-hexane solution. The product was purified by dissolving in methanolic solution containing small volume of triethylamine. The process repeated two times to get pure recrystalised dicoumarol ligand. Yield: 70%; m.p. 114°C. IR ʋ (cm^-1^): 3078 (OH); 1647 (CO); 1562 (C = C); 1303 (Lactone C-O). ^1^H NMR (300 MHz, DMSO-*d*^*6*^) (δ, ppm): 6.10 (s, 1H, H12); 7.22-7.67 (m, 11H, H-arom); 11.34 (s, 2H, OH). ^13^C {^1^H}-NMR (75.47 MHz, DMSO-*d*^*6*^) (δ, ppm): 36 (C12); 102 (C3, 13); 106 (C27); 116 (C9, 21); 117 (C8, 20); 121 (C26, 28); 123 (C24); 127 (C7, 19); 135 (C4, 18); 152 (C6, 16); 164 (C2, 14). MS, m/ z (%): 413.1022 [C_25_H_16_O_6_ + H]^+^ (100), Elemental Analysis (C_25_H_16_O_6_), Calc. C: 72.81%, H:3.91% Exp. C: 72.83, H: 3.88, Λ_m_: 240 μS.

#### Synthesis of sodium compound of 4-Hydroxy-3-[(4-hydroxy-2-oxo-4a,8a-dihydro-2H-chromen-3-yl)-phenyl-methyl]-chromen-2-one (1)

10 mmol of the dicoumarol compound was added to 20 cm^3^ methanol containing 25 mmol of sodium methoxide. The mixture was stirred for about an hour and left over night. Next day transparent block golden X-ray quality single crystals were isolated. Yield: 63%; IR ʋ (cm^-1^): 3658 (OH); 1671 (CO); 1534 (C = C); 1349 (Na-O lactone). ^1^H NMR (400.23 MHz, DMSO-*d*^*6*^, 303 k) (δ, ppm): 2.52 (s, 12H, Na-OCH_3_); 6.10 (s, 2H, C6); 7.11-7.67 (m, 26 H, Harom); 8.31 (s, 2H, OH); ^13^C {^1^H}-NMR (150.9 MHz, DMSO-*d*^*6*^, 303 k) (δ, ppm): 33 (C6); 103 (C5, 7); 106 (C19); 116 (C13, 23); 117 (C14, 23); 121 (C18, 20); 122 (C17, 21); 123 (C16); 131 (C15, 25); 145 (C4, 11); 164 (C2, 9); 167 (C1, 8). ^23^Na-NMR (400.23 MHz, DMSO-*d*^*6*^, 303 k) (δ, ppm): -1.01. MS, m/ z (%): No molecular ion peak observed. Elemental Analysis (C_54_H_44_Na_2_O_16_), Calc. C: 65.19%, H: 4.46% Exp. C: 65.23%, H: 4.23, Λ_m_: 410 μS.

## Conclusions

A sodium analogue (**1**) with medicinally important dicoumarol ligand (**L**) has been reported. Spectral analysis and X-ray crystallographic techniques have been used to determine structure of the compound. The crystal structure and DFT study confirm the formation of cationic sodium compound with dicoumarol. The ligand was found more active than the sodium analog attributed to the instability of **1** in solution state. Coumarin compound with sodium was observed to be less cytotoxic than the ligand, its LD_50_ value never dropped below 60%.

## Competing interests

The authors declare that they have no competing interests.

## Authors’ contributions

SR and MI carried out the original laboratory work in the lab. of RJB under the supervision of SUR. EA, SM and KHM analysed the samples for their U2OS anticancer studies. AK and TSH carried out the theoretical studies. The structure was solved by SR and AJB. All authors read and approved the final manuscript.

## Supplementary Material

Additional file 1 Supporting Information. Click here for file
