# Supplementary material for: New dicoumarol sodium compound: crystal structure, theoretical study and tumoricidal activity against osteoblast cancer cells
Source: Chem Cent J. 2013 Jul 2;7:110. doi: 10.1186/1752-153X-7-110 (PMC3716688; doi:10.1186/1752-153X-7-110)
Supplement: Additional file 1 — Supporting Information. [file 1752-153X-7-110-S1.doc]

New dicoumarol sodium compound: Crystal structure, theoretical study and tumoricidal activity against osteoblast cancer cells

Sadia Rehman, Muhammad Ikram, Ajmal Khan, Soyoung Min, Effat Azad, Thomas S. Hofer, M. Iqbal Choudhary, K. H. Mok, Robert J. Baker, Alexander J. Blake and Saeed-Ur- Rehman

***Supporting Information***

1. Experimental Section ………………………………………… 2

A.1 ^1^H-NMR of 1 ……………………………………………. 3

A.2 ^13^C-NMR of 1 …………………………………………… 4

A.3 ^23^Na-NMR of 1………………………………………….. 5

B. Crystal Data of 1 …………………………………………………. 5

B.1 Crystal structure of 1 …………………………………… 10

B.2 Space filled structure of 1 ……………………………… 11

C. X,Y, and Z coordinates of 1 …………………………………….. 12

C.1 Theoretical Structures of 1 …………………………….. 19

C.2 Iso-density surface diagram of 1 ………………………. 19

D. References ………………………………………………………. 20

A. Experimental Section

All the manipulations were performed under argon environment using Schlenk line system. 4-hydroxycoumarine (fluka), benzaldehyde and benazldehyde (Sigma Aldrich) were used as received. Analytical grade methanol (Aldrich) was dehydrated and degassed prior to use. Sodium metal (Fluka) was added to methanol to produce sodium methoxide under argon. Elemental analyses were carried out on Varian Elementar II. FT-IR spectra were recorded using Shimadzo FTIR Spectrophotometer Prestige-21. ^1^H-NMR were measured with Bruker DPX 400MHz (400.23 MHz) whereas, ^13^C{^1^H}NMR were recorded on Bruker AV 400MHz (150.9 MHz) spectrometers in d^6^-DMSO at room temperature. Chemical shifts are reported in ppm and standardized by observing signals for residual protons. Mass spectra were recorded on a LCT Orthogonal Acceleration TOF Electrospray mass spectrometer. Single crystal analyses were carried out using . Suitable single crystals for X-ray structural analyses of 1 was mounted on a glass fibre, and the respective data were collected on Oxford diffractometer (graphite-monochromated Mo Kα radiation, λ = 0.71073 Å) at 108(2) K. The structures were solved with the olex2.solve ^1^ structure solution program using Charge Flipping and refined with the olex2.refine ^2^ refinement package using Gauss-Newton minimisation. Crystallographic details are given in the supplementary information file. CCDC-868924

(1) data can be obtained free of charge from the Cambridge Crystallographic Data Centre via [www.ccdc.cam.ac.uk/data_request/cif](http://www.ccdc.cam.ac.uk/data_request/cif).

The geometry optimization was carried out at the restricted B3LYP hybrid density functional level, as implemented in the G09 program. ^3,4^ The x-ray structure was employed as starting configuration for the geometry optimization. The 6-31g(d,p) basis set was assigned to all atoms.^5^

The dicoumarile ligand (L) was synthesized by the reported procedure , 25mmol benazldehyde was added to the 50mmol stirred ethanolic solution of 4-hydroxycoumarine and the mixture was refluxed for 3hr at 120^o^C.^6^ After cooling the reaction mixture solid white powder of the L were isolated, washed several times with copious 10% ethanolic - n-hexane solution. The product was purified by dissolving in methanolic solution containing small volume of triethylamine. The process repeated tw times to get pure recrystalised dicoumarile ligand. Sodium adduct of the dicoumarile ligand was isolated unexpectedly. Actually efforts were done to isolate the sodium salt of dicoumarile ligand after deprotonating the hydroxyl groups of the coumarine rings as shown in scheme 1. 10mmol of the dicoumarile compound was added to 20cm^3^ methanol containing 25mmol of sodium methoxide. The mixture was stirred for about an hour and left forth night. Next day transparent block golden x-ray quality single crystals were isolated. The compound turns white powder upon the addition of THF.

Cytotoxic activity: The human osteosarcoma cell line U2OS was used for testing the tumoricidal activities of the 1 and L compounds. The cells were cultured in DMEM+ GlutaMAXTM-1 with added 1% penicillin and streptomycin and 10% heat-inactivated fetal bovine serum. For adherent cells, trypsin-EDTA was used for detachment. The cells were washed in Dulbecco’s phosphate buffered saline (DPBS), harvested by centrifugation (1000 rpm, 5 min), and resuspended in DMEM. The cells were seeded into 96-well plates at a density of 5x10^3^ cells / well, and incubated at 37 °C in 5% CO2 atmosphere for 24 hours before being treated with the compounds. The compounds were initially dissolved at a stock concentration of 10 mM in DMSO and added to wells to a final concentration between 5.0 and 2000 μM. The plates were then incubated at 37 °C for a further 24 hours for treatment. Alamar blue (Invitrogen) was used to test the viability of the cells (10 μl per well). Plates in triplicate were incubated for 4 hours at 37 °C protected from direct light, then read at 590 nm using an excitation wavelength of 544 nm in a fluorescence plate reader (Spectra Max Gemini). Wells containing medium and distilled water-only served as blank controls, while the viability of the treated cells was taken as a percentage compared to wells with untreated cells. The LD50 value of each compound was estimated by fitting the correlation between cell viability and compound concentration.

A.1 ^1^H-NMR of 1

A.2 ^13^C-NMR of 1

A.3 ^23^Na-NMR of 1

B. Crystal Data of 1

| Table 1 Crystal data and structure refinement for 1 | |
| --- | --- |
| Empirical formula | C_54_H_44_Na_2_O_16_ |
| Formula weight | 994.89 |
| Temperature/K | 298 |
| Crystal system | monoclinic |
| Space group | P2_1_/n |
| a/Å | 12.3792(14) |
| b/Å | 11.5396(7) |
| c/Å | 16.980(2) |
| α/° | 90.0 |
| β/° | 102.354(12) |
| γ/° | 90.0 |
| Volume/Å^3^ | 2369.4(4) |
| Z | 1 |
| ρ_calc_ g/cm^3^ | 1.389 |
| m/mm^‑1^ | 0.118 |
| F(000) | 1028 |
| Crystal size/mm^3^ | N/A × N/A × N/A |
| 2Θ range for data collection | 6.04 to 53.88° |
| Index ranges | -15 ≤ h ≤ 14, 0 ≤ k ≤ 13, 0 ≤ l ≤ 21 |
| Reflections collected | 7498 |
| Independent reflections | 4233[R(int) = 0.1007] |
| Data/restraints/parameters | 4233/2/334 |
| Goodness-of-fit on F^2^ | 1.072 |
| Final R indexes [I>=2σ (I)] | R_1_ = 0.1163, wR_2_ = N/A |
| Final R indexes [all data] | R_1_ = 0.2101, wR_2_ = 0.2004 |
| Largest diff. peak/hole / e Å^-3^ | 0.96/-0.80 |

| Table 2 Fractional Atomic Coordinates (×10^4^) and Equivalent Isotropic Displacement Parameters (Å^2^×10^3^) for 1. U_eq_ is defined as 1/3 of of the trace of the orthogonalised U_IJ_ tensor. | | | | |
| --- | --- | --- | --- | --- |
| Atom | *x* | *y* | *z* | U(eq) |
| Na | 4199(2) | 5251.1(19) | 5708.2(17) | 53.0(8) |
| O | 6005(4) | 4425(4) | 5758(3) | 51.0(13) |
| O1aa | 7401(4) | 3488(3) | 5454(3) | 45.2(12) |
| O3aa | 7017(4) | 1904(3) | 8509(3) | 45.1(12) |
| O0aa | 8444(4) | 2312(3) | 7758(3) | 42.4(12) |
| O5aa | 3819(4) | 2817(3) | 7471(3) | 49.6(13) |
| C8 | 4663(6) | 3553(5) | 7362(4) | 40.5(17) |
| C10 | 7250(6) | 4608(4) | 8968(4) | 45.4(19) |
| C11 | 10135(7) | 1382(5) | 5951(5) | 60(2) |
| C12 | 7229(5) | 3501(4) | 6839(4) | 33.5(15) |
| C13 | 8337(6) | 2804(4) | 5661(5) | 42.5(18) |
| C15 | 5098(6) | 1535(4) | 8300(4) | 40.0(17) |
| C16 | 8688(5) | 2392(4) | 6415(4) | 35.1(16) |
| C17 | 7482(6) | 4537(4) | 8204(4) | 37.5(16) |
| C18 | 9751(7) | 1798(6) | 5189(5) | 64(2) |
| C19 | 6841(6) | 3828(5) | 6039(4) | 41.7(18) |
| C20 | 6019(6) | 2255(4) | 8165(4) | 36.5(16) |
| C21 | 5281(6) | 509(5) | 8766(5) | 50(2) |
| C22 | 8831(7) | 2530(5) | 5012(5) | 54(2) |
| C23 | 8428(6) | 5070(4) | 8080(4) | 46.3(19) |
| C24 | 5779(5) | 3229(4) | 7684(4) | 35.6(16) |
| C25 | 4067(6) | 1817(5) | 7919(5) | 45.9(19) |
| C26 | 4372(7) | -184(5) | 8805(6) | 66(3) |
| C27 | 8122(5) | 2725(4) | 7043(4) | 34.9(16) |
| C28 | 6668(5) | 4023(4) | 7481(4) | 34.7(15) |
| C29 | 7921(7) | 5188(5) | 9575(5) | 56(2) |
| C30 | 9628(6) | 1670(5) | 6569(5) | 49.6(19) |
| C31 | 9120(7) | 5658(5) | 8704(5) | 60(2) |
| C32 | 3136(6) | 1102(5) | 7938(5) | 55(2) |
| C33 | 3324(7) | 89(5) | 8382(5) | 63(2) |
| C34 | 8875(7) | 5731(5) | 9453(5) | 58(2) |
| O1 | 3057(5) | 3693(4) | 5140(4) | 78.3(18) |
| O2 | 4486(5) | 7105(4) | 6293(4) | 79.1(18) |
| O2aa | 4354(4) | 4419(3) | 6983(3) | 50.7(13) |
| C1 | 3900(8) | 8088(6) | 6501(7) | 94(3) |
| C2 | 3101(15) | 2645(8) | 5440(7) | 185(8) |

| Table 3 Anisotropic Displacement Parameters (Å^2^×10^3^) for 1. The Anisotropic displacement factor exponent takes the form: -2π^2^[h^2^a*^2^U_11_+...+2hka×b×U_12_] | | | | | | |
| --- | --- | --- | --- | --- | --- | --- |
| Atom | U_11_ | U_22_ | U_33_ | U_12_ | U_13_ | U_23_ |
| Na | 45(2) | 51.6(13) | 61.2(19) | 2.5(12) | 7.8(16) | 6.0(12) |
| O | 37(4) | 68(3) | 46(3) | 10(2) | 2(3) | 13(2) |
| O1aa | 40(4) | 50(2) | 47(3) | 1(2) | 10(3) | 7(2) |
| O3aa | 34(4) | 38(2) | 66(3) | 0.2(19) | 16(3) | 13(2) |
| O0aa | 28(3) | 41(2) | 56(3) | 7.5(18) | 5(2) | 10(2) |
| O5aa | 24(3) | 58(2) | 66(3) | 2(2) | 8(3) | 17(2) |
| C8 | 23(5) | 52(3) | 47(4) | 4(3) | 9(4) | 12(3) |
| C10 | 50(6) | 35(3) | 52(5) | -1(3) | 14(4) | 0(3) |
| C11 | 60(7) | 53(4) | 74(6) | 9(3) | 26(5) | -7(4) |
| C12 | 23(4) | 33(3) | 45(4) | -5(2) | 8(3) | 7(3) |
| C13 | 33(5) | 35(3) | 60(5) | -4(3) | 12(4) | -8(3) |
| C15 | 29(5) | 34(3) | 60(5) | 1(3) | 14(4) | 3(3) |
| C16 | 28(5) | 29(2) | 50(4) | -2(2) | 12(4) | 1(3) |
| C17 | 31(5) | 31(3) | 47(4) | 6(3) | 1(4) | 8(3) |
| C18 | 63(7) | 66(4) | 69(6) | 2(4) | 28(5) | -14(4) |
| C19 | 36(5) | 36(3) | 54(5) | -5(3) | 11(4) | 2(3) |
| C20 | 28(5) | 37(3) | 45(4) | 2(3) | 10(4) | 0(3) |
| C21 | 43(6) | 43(3) | 70(5) | 1(3) | 23(5) | 7(3) |
| C22 | 47(6) | 52(4) | 68(6) | -9(3) | 21(5) | -11(4) |
| C23 | 38(5) | 38(3) | 60(5) | -5(3) | 5(4) | 3(3) |
| C24 | 23(5) | 33(3) | 52(4) | 1(2) | 10(4) | 4(3) |
| C25 | 31(5) | 42(3) | 66(5) | -2(3) | 14(4) | 3(3) |
| C26 | 49(6) | 41(3) | 116(7) | -3(3) | 38(6) | 12(4) |
| C27 | 26(4) | 28(2) | 50(4) | -3(2) | 5(4) | 1(3) |
| C28 | 25(4) | 33(3) | 46(4) | -1(2) | 7(4) | 4(3) |
| C29 | 78(7) | 37(3) | 55(5) | 2(3) | 16(5) | -2(3) |
| C30 | 43(5) | 40(3) | 68(5) | -2(3) | 14(4) | -7(3) |
| C31 | 47(6) | 49(4) | 80(6) | -9(3) | 6(5) | -6(4) |
| C32 | 26(5) | 59(4) | 80(6) | -5(3) | 12(4) | 1(4) |
| C33 | 40(6) | 52(4) | 102(7) | -7(3) | 26(5) | 4(4) |
| C34 | 52(6) | 39(3) | 72(6) | 4(3) | -12(5) | -5(3) |
| O1 | 91(6) | 52(3) | 88(4) | -10(3) | 9(4) | 3(3) |
| O2 | 57(5) | 68(3) | 103(5) | -1(3) | -3(4) | -11(3) |
| O2aa | 28(3) | 57(2) | 63(3) | 11(2) | 2(3) | 18(2) |
| C1 | 59(8) | 68(5) | 149(10) | 3(4) | 11(7) | 15(5) |
| C2 | 340(20) | 65(6) | 106(10) | -25(9) | -52(12) | 6(6) |

| Table 4 Bond Lengths for 1. | | | | | | |
| --- | --- | --- | --- | --- | --- | --- |
| Atom | Atom | Length/Å |  | Atom | Atom | Length/Å |
| Na | Na^1^ | 3.477(5) |  | C13 | C16 | 1.348(9) |
| Na | O | 2.415(5) |  | C13 | C22 | 1.406(9) |
| Na | O^1^ | 2.476(5) |  | C15 | C20 | 1.468(8) |
| Na | O1 | 2.362(6) |  | C15 | C21 | 1.416(8) |
| Na | O2 | 2.354(5) |  | C15 | C25 | 1.342(10) |
| Na | O2aa | 2.338(5) |  | C16 | C27 | 1.448(8) |
| O | C19 | 1.250(8) |  | C16 | C30 | 1.410(9) |
| O1aa | C13 | 1.384(8) |  | C17 | C23 | 1.378(9) |
| O1aa | C19 | 1.384(7) |  | C17 | C28 | 1.532(9) |
| O3aa | C20 | 1.313(8) |  | C18 | C22 | 1.398(11) |
| O0aa | C27 | 1.286(7) |  | C20 | C24 | 1.384(8) |
| O5aa | C8 | 1.389(7) |  | C21 | C26 | 1.394(9) |
| O5aa | C25 | 1.381(7) |  | C23 | C31 | 1.389(10) |
| C8 | C24 | 1.422(9) |  | C24 | C28 | 1.527(7) |
| C8 | O2aa | 1.206(7) |  | C25 | C32 | 1.423(9) |
| C10 | C17 | 1.390(8) |  | C26 | C33 | 1.379(11) |
| C10 | C29 | 1.354(10) |  | C29 | C34 | 1.391(10) |
| C11 | C18 | 1.366(11) |  | C31 | C34 | 1.372(10) |
| C11 | C30 | 1.374(9) |  | C32 | C33 | 1.384(10) |
| C12 | C19 | 1.392(9) |  | O1 | C2 | 1.309(10) |
| C12 | C27 | 1.407(8) |  | O2 | C1 | 1.430(9) |
| C12 | C28 | 1.535(8) |  |  |  |  |

^1^1-X,1-Y,1-Z

| Table 5 Bond Angles for 1. | | | | | | | | |
| --- | --- | --- | --- | --- | --- | --- | --- | --- |
| Atom | Atom | Atom | Angle/˚ |  | Atom | Atom | Atom | Angle/˚ |
| O1 | Na | O^1^ | 77.31(19) |  | C22 | C18 | C11 | 121.3(7) |
| O1 | Na | O | 100.5(2) |  | O1aa | C19 | O | 112.5(6) |
| O2 | Na | O^1^ | 105.1(2) |  | C12 | C19 | O | 127.4(6) |
| O2 | Na | O | 106.8(2) |  | C12 | C19 | O1aa | 120.2(6) |
| O2 | Na | O1 | 152.5(2) |  | C15 | C20 | O3aa | 116.4(5) |
| O2aa | Na | O | 85.05(17) |  | C24 | C20 | O3aa | 125.2(5) |
| O2aa | Na | O^1^ | 164.42(18) |  | C24 | C20 | C15 | 118.4(6) |
| O2aa | Na | O1 | 89.39(19) |  | C26 | C21 | C15 | 118.2(7) |
| O2aa | Na | O2 | 90.4(2) |  | C18 | C22 | C13 | 115.9(8) |
| C19 | O | Na^1^ | 111.5(4) |  | C31 | C23 | C17 | 120.4(7) |
| C19 | O | Na | 156.3(4) |  | C20 | C24 | C8 | 120.5(5) |
| C19 | O1aa | C13 | 120.0(5) |  | C28 | C24 | C8 | 116.3(5) |
| C25 | O5aa | C8 | 119.9(6) |  | C28 | C24 | C20 | 123.1(6) |
| C24 | C8 | O5aa | 119.0(5) |  | C15 | C25 | O5aa | 123.0(6) |
| O2aa | C8 | O5aa | 114.5(6) |  | C32 | C25 | O5aa | 114.3(7) |
| O2aa | C8 | C24 | 126.4(6) |  | C32 | C25 | C15 | 122.7(6) |
| C29 | C10 | C17 | 121.6(6) |  | C33 | C26 | C21 | 121.6(7) |
| C30 | C11 | C18 | 121.0(7) |  | C12 | C27 | O0aa | 122.5(5) |
| C27 | C12 | C19 | 120.1(6) |  | C16 | C27 | O0aa | 119.7(5) |
| C28 | C12 | C19 | 118.2(5) |  | C16 | C27 | C12 | 117.8(6) |
| C28 | C12 | C27 | 121.7(6) |  | C17 | C28 | C12 | 113.7(5) |
| C16 | C13 | O1aa | 121.6(6) |  | C24 | C28 | C12 | 112.5(4) |
| C22 | C13 | O1aa | 114.2(7) |  | C24 | C28 | C17 | 115.7(5) |
| C22 | C13 | C16 | 124.0(7) |  | C34 | C29 | C10 | 120.7(7) |
| C21 | C15 | C20 | 121.6(6) |  | C16 | C30 | C11 | 119.7(7) |
| C25 | C15 | C20 | 118.6(5) |  | C34 | C31 | C23 | 121.0(7) |
| C25 | C15 | C21 | 119.6(6) |  | C33 | C32 | C25 | 117.3(8) |
| C27 | C16 | C13 | 119.9(6) |  | C32 | C33 | C26 | 120.5(7) |
| C30 | C16 | C13 | 118.0(6) |  | C31 | C34 | C29 | 118.3(7) |
| C30 | C16 | C27 | 122.1(6) |  | C2 | O1 | Na | 124.9(7) |
| C23 | C17 | C10 | 118.0(6) |  | C1 | O2 | Na | 141.8(5) |
| C28 | C17 | C10 | 122.6(6) |  | C8 | O2aa | Na | 142.0(4) |
| C28 | C17 | C23 | 119.0(6) |  |  |  |  |  |

^1^1-X,1-Y,1-Z

| Table 6 Hydrogen Atom Coordinates (Å×10^4^) and Isotropic Displacement Parameters (Å^2^×10^3^) for 1. | | | | |
| --- | --- | --- | --- | --- |
| Atom | *x* | *y* | *z* | U(eq) |
| H3a | 7453(12) | 2100(60) | 8230(30) | 67.7(18) |
| H10 | 6619(6) | 4248(4) | 9065(4) | 54(2) |
| H11 | 10749(7) | 897(5) | 6053(5) | 73(3) |
| H18 | 10107(7) | 1589(6) | 4781(5) | 77(3) |
| H21 | 5989(6) | 302(5) | 9039(5) | 61(2) |
| H23 | 8605(6) | 5035(4) | 7574(4) | 56(2) |
| H26 | 4475(7) | -847(5) | 9125(6) | 79(3) |
| H28 | 6259(5) | 4693(4) | 7215(4) | 41.7(18) |
| H29 | 7742(7) | 5224(5) | 10080(5) | 68(3) |
| H30 | 9904(6) | 1390(5) | 7087(5) | 60(2) |
| H31 | 9758(7) | 6008(5) | 8612(5) | 72(3) |
| H32 | 2429(6) | 1308(5) | 7662(5) | 66(2) |
| H33 | 2739(7) | -411(5) | 8395(5) | 76(3) |
| H34 | 9334(7) | 6133(5) | 9868(5) | 70(3) |
| H1a | 3180(30) | 8130(40) | 6150(30) | 141(5) |
| H1b | 3820(60) | 8020(40) | 7048(17) | 141(5) |
| H1c | 4300(40) | 8782(9) | 6450(50) | 141(5) |
| H2a | 2400(40) | 2270(50) | 5260(90) | 277(12) |
| H2b | 3670(100) | 2210(50) | 5270(90) | 277(12) |
| H2c | 3270(150) | 2688(10) | 6018(7) | 277(12) |
| H2 | 5140(70) | 7070(110) | 6580(90) | 277(12) |
| H22 | 8460(70) | 2860(60) | 4270(50) | 50(20) |

B.1 Crystal structure of 1


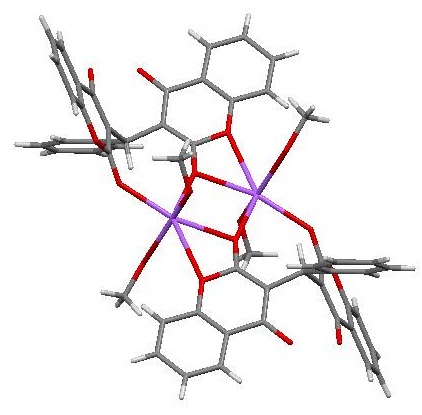


B.2 Space filled structure of 1


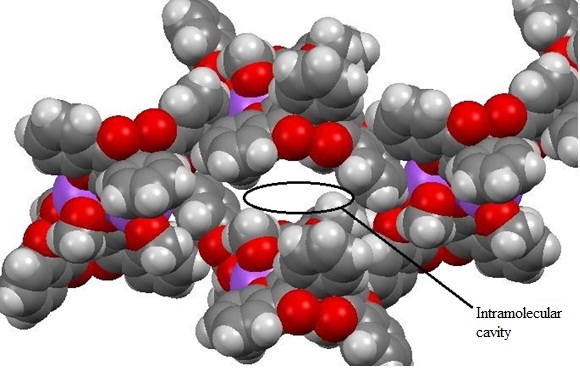


C. X,Y, and Z coordinates of 1

116 Min-Geometry of Sodide complex

B3LYP/6-31G(d,p) Energy: -3612.21348435 Hartree

Na 0.743199 1.629476 -0.550621

O -1.326018 0.423082 -0.653043

O -2.285344 2.267551 0.070325

O -4.700095 1.697260 -2.875767

O -6.592713 0.806078 -0.757020

O -4.735681 -0.820392 2.506208

O -2.754316 -0.718749 1.571488

O 1.473103 3.062261 1.104591

O 0.452912 3.184232 -2.220306

C -2.366984 1.045195 -0.489350

C -3.360003 3.184154 0.017847

C -4.397188 3.004452 -0.899851

C -4.393365 1.763313 -1.709871

C -3.741653 0.597364 -0.917550

C -4.024906 -0.845280 -1.099409

H -3.185193 -1.470366 -0.797751

C -4.604983 -0.115681 0.169079

C -3.958501 -0.548332 1.446086

C -6.124239 -0.674309 2.501964

C -6.810278 -0.168362 1.393739

C -6.076017 0.232739 0.188842

C -8.206833 -0.027582 1.472930

H -8.729755 0.370467 0.610111

C -8.881407 -0.397412 2.628599

H -9.959434 -0.294584 2.685901

C -8.166636 -0.902197 3.723311

H -8.693074 -1.188058 4.628103

C -6.779635 -1.041452 3.669748

H -6.210909 -1.422163 4.510400

C -4.903418 -1.507734 -2.120776

C -4.767780 -1.248927 -3.490033

H -4.113590 -0.455687 -3.828663

C -5.501899 -1.990023 -4.417508

H -5.398082 -1.773585 -5.475991

C -6.372667 -2.992520 -3.991060

H -6.947834 -3.559766 -4.715832

C -6.507037 -3.259903 -2.626994

H -7.186157 -4.035173 -2.286558

C -5.770663 -2.526881 -1.698490

H -5.881595 -2.741029 -0.638102

C -5.369144 4.002833 -1.025178

H -6.169692 3.860993 -1.743199

C -5.308105 5.134400 -0.214424

H -6.070183 5.901572 -0.297615

C -4.266724 5.282103 0.707551

H -4.221674 6.164951 1.336720

C -3.269614 4.309069 0.823594

H -2.440640 4.421685 1.513641

C 1.974564 4.399058 0.906356

H 3.031339 4.468388 1.182929

H 1.396611 5.128193 1.482681

H 1.862870 4.614061 -0.157139

C 1.073944 3.134300 -3.521250

H 0.343787 2.888512 -4.299141

H 1.829220 2.349971 -3.471485

H 1.558116 4.086010 -3.762010

Na -0.748196 -1.555417 0.733846

O 1.351453 -0.382197 0.793491

O 2.216401 -2.208282 -0.078838

O 4.782221 -1.878142 2.762237

O 6.603660 -0.903573 0.616638

O 4.652961 0.940865 -2.470079

O 2.705966 0.799454 -1.469938

O -1.437223 -3.110509 -0.834521

O -0.480990 -3.021341 2.487405

C 2.363753 -1.020760 0.538856

C 3.266811 -3.151372 -0.140935

C 4.357115 -3.050957 0.725772

C 4.425445 -1.861864 1.608833

C 3.772120 -0.633933 0.917048

C 4.099610 0.791402 1.155478

H 3.260895 1.446572 0.922396

C 4.601445 0.107144 -0.174281

C 3.912535 0.614253 -1.398896

C 6.038674 0.778539 -2.527066

C 6.757889 0.196815 -1.478666

C 6.062694 -0.265804 -0.272904

C 8.148106 0.042575 -1.619565

H 8.696754 -0.413864 -0.802873

C 8.784572 0.473338 -2.775773

H 9.857961 0.359951 -2.880532

C 8.037245 1.053664 -3.809588

H 8.533849 1.387260 -4.714906

C 6.655535 1.207682 -3.694693

H 6.061769 1.646120 -4.488601

C 5.046911 1.391765 2.153000

C 5.036016 1.032763 3.506014

H 4.423566 0.206314 3.841981

C 5.841040 1.719009 4.417242

H 5.833022 1.425553 5.462147

C 6.658864 2.764942 3.991351

H 7.288850 3.289198 4.702803

C 6.668889 3.132361 2.643785

H 7.305815 3.942544 2.303041

C 5.862084 2.454705 1.732560

H 5.876489 2.746050 0.684802

C 5.308085 -4.076926 0.737024

H 6.150226 -3.997182 1.415926

C 5.172735 -5.155757 -0.134490

H 5.917910 -5.943738 -0.139817

C 4.078110 -5.224258 -1.002611

H 3.975119 -6.066789 -1.678496

C 3.102150 -4.223454 -1.004495

H 2.232754 -4.275499 -1.650462

C -1.969639 -4.416530 -0.535803

H -3.022647 -4.488427 -0.825911

H -1.396760 -5.201540 -1.039159

H -1.879782 -4.544790 0.543544

C -1.115700 -2.894916 3.776561

H -0.395950 -2.592556 4.544135

H -1.878367 -2.123592 3.669198

H -1.592918 -3.834332 4.072889

H -1.474337 -2.973984 -1.789992

H -0.199328 3.895912 -2.222237

H 1.528752 2.847558 2.044668

H 0.175304 -3.727424 2.540061

C.1 Theoretical Structures of 1


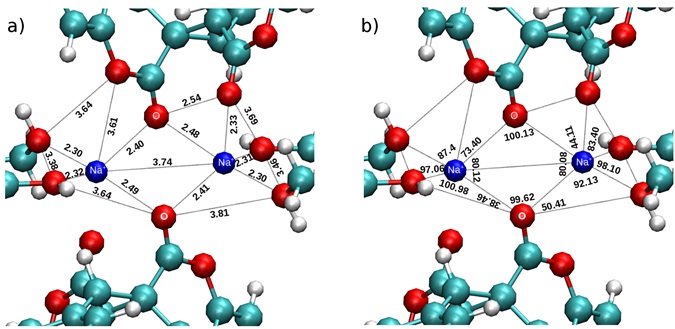


C.2 Iso-density surface diagram of 1


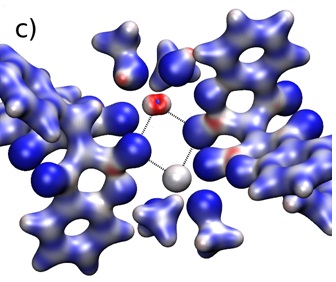


**References**

1. O. V. Dolomanov, L. J. Bourhis, R. J. Gildea, J. A. K. Howard and H. Puschmann, OLEX2: a complete structure solution, refinement and analysis program. J. Appl. Cryst. (2009). 42, 339-341.
2. olex2.refine (L.J. Bourhis, O.V. Dolomanov, R.J. Gildea, J.A.K. Howard, H. Puschmann, in preparation, 2011)
3. A. D. Becke, *J. Chem. Phys.* **1993**, *98*, 5648-5652
4. M. J. Frisch, G. W. Trucks, H. B. Schlegel, G. E. Scuseria, M. A. Robb, J. R. Cheeseman, G. Scalmani, V. Barone, B. Mennucci, G. A. Petersson, H. Nakatsuji, M. Caricato, X. Li, H. P. Hratchian, A. F. Izmaylov, J. Bloino, G. Zheng, J. L. Sonnenberg, M. Hada, M. Ehara, K. Toyota, R. Fukuda, J. Hasegawa, M. Ishida, T. Nakajima, Y. Honda, O. Kitao, H. Nakai, T. Vreven, J. A. J. Montgomery, J. E. Peralta, F. Ogliaro, M. Bearpark, J. J. Heyd, E. Brothers, K. N. Kudin, V. N. Staroverov, R. Kobayashi, J. Normand, K. Raghavachari, A. Rendell, J. C. Burant, S. S. Iyengar, J. Tomasi, M. Cossi, N. Rega, J. M. Millam, M. Klene, J. E. Knox, J. B. Cross, V. Bakken, C. Adamo, J. Jaramillo, R. Gomperts, R. E. Stratmann, O. Yazyev, A. J. Austin, R. Cammi, C. Pomelli, J. W. Ochterski, R. L. Martin, K. Morokuma, V. G. Zakrzewski, G. A. Voth, P. Salvador, J. J. Dannenberg, S. Dapprich, A. D. Daniels, O. Farkas, J. B. Foresman, J. V. Ortiz, J. Cioslowski and D. J. Fox, *Gaussian 09, Revision A.02. Gaussian, Inc., Wallingford, CT,* **2009**.
5. W. J. Hehre, R. Ditchfield and J. A. Pople, *J. Chem. Phys.,* **1972**, *56*, 2257–2261.
6. N. Hamdi, M. C. Puerta, P. Valerga, *Eur. J. Med. Chem.,* **2008**, *43*, 2541-2548
